# Emotional status and fear in patients scheduled for elective surgery during COVID-19 pandemic: a nationwide cross-sectional survey (COVID-SURGERY)

**DOI:** 10.1186/s44158-021-00022-7

**Published:** 2021-11-25

**Authors:** Francesca Montalto, Mariachiara Ippolito, Alberto Noto, Fabiana Madotto, Filippa Gelardi, Paolino Savatteri, Antonino Giarratano, Andrea Cortegiani, the SIAARTI Study Group, Fabrizio Brescia, Fabio Fabiani, Chiara Zanier, Elisa Nadalini, Eros Gambaretti, Francesco Gabriele, Marinella Astuto, Paolo Murabito, Filippo Sanfilippo, Giovanni Misseri, Alessandra Moscarelli, Savino Spadaro, Enrico Bussolati, Eleonora Squadrani, Gianluca Villa, Raffaella D’Errico, Giulia Cocci, Iacopo Lanini, Lucia Mirabella, Alessandra Morelli, Livio Tullo, Girolamo Caggianelli, Lorenzo Ball, Margherita Iiriti, Francesca Giordani, Massimiliano Giardina, Anna Teresa Mazzeo, Giacomo Grasselli, Emanuele Cattaneo, Salvatore Alongi, Cristina Marenghi, Marilena Marmiere, Margherita Rocchi, Stefano Turi, Giovanni Landoni, Vito Torrano, Giulia Tinti, Antonio Giorgi, Roberto Fumagalli, Francesco Salvo, Ilaria Blangetti, Marco Cascella, Cira Antonietta Forte, Paolo Navalesi, Marta Montalbano, Valentina Chiarelli, Giuseppe Bonanno, Francesco Paolo Ferrara, Innocenza Pernice, Giulia Catalisano, Claudia Marino, Gabriele Presti, Dario Calogero Fricano, Rosa Fucà, Cesira Palmeri di Villalba, Maria Teresa Strano, Sabrina Caruso, Antonino Scafidi, Vincenzo Mazzarese, Ettore Augugliaro, Valeria Terranova, Francesco Forfori, Francesco Corradi, Erika Taddei, Alessandro Isirdi, Giorgia Pratesi, Francesca Puccini, Gianluca Paternoster, Alessio Barile, Marco Tescione, Irene Santacaterina, Eliana Maria Siclari, Vincenzo Francesco Tripodi, Mariacristina Vadalà, Felice Eugenio Agrò, Giuseppe Pascarella, Chiara Piliego, Paola Aceto, Gennaro De Pascale, Alessandra Dottarelli, Bruno Romanò, Andrea Russo, Marco Covotta, Valeria Giorgerini, Federica Sardellitti, Giulia Maria Vitelli, Flaminia Coluzzi, Tiziana Bove, Luigi Vetrugno

**Affiliations:** 1 UOC Anestesia Rianimazione1 PO Villa Sofia AOOR Villa Sofia-Cervello, Palermo, Italy; 2 Department of Surgical Oncological and Oral Science (Di.Chir.On.S.), University of Palermo, Palermo, Italy; 3 Department of Human Pathology of the Adult and Evolutive Age “Gaetano Barresi”, Division of Anesthesia and Intensive Care, University of Messina, Messina, Italy; 4 Value-Based Healthcare Unit, IRCCS Multimedica, 20099 Sesto San Giovanni, Milan, Italy; 5 Freelance Psychologist, Milan, Italy; 6 Department of Anesthesia, Intensive Care and Emergency, Policlinico Paolo Giaccone, Palermo, Italy

**Keywords:** COVID-19, Perioperative medicine, Emotional status, Survey

## Abstract

**Background:**

Fragmented data exist on the emotional and psychological distress generated by hospital admission during the pandemic in specific populations of patients, and no data exists on patients scheduled for surgery. The aim of this multicentre nationwide prospective cross-sectional survey was to evaluate the impact of pandemic on emotional status and fear of SARS-CoV-2 contagion in a cohort of elective surgical patients in Italy, scheduled for surgery during the COVID-19 pandemic.

**Results:**

Twenty-nine Italian centres were involved in the study, for a total of 2376 patients surveyed (mean age of 58 years ± 16.61; 49.6% males). The survey consisted of 28 total closed questions, including four study outcome questions. More than half of patients had at least one chronic disease (54%), among which cardiovascular diseases were the commonest (58%). The most frequent type of surgery was abdominal (20%), under general anaesthesia (64%). Almost half of the patients (46%) declared to be frightened of going to the hospital for routine checkups; 55% to be afraid of getting SARS-CoV-2 infection during hospitalization and 62% were feared of being hospitalised without seeing family members. Having an oncological disease and other patient-related, centre-related or perioperative factors were independently associated with an increased risk of fear of SARS-CoV-2 infection during hospitalization and of being hospitalised without seeing family members. A previous infection due to SARS-COV-2 was associated with a reduced risk of worse emotional outcomes and fear of SARS-CoV-2 infection during hospitalization. Patients who showed the most emotionally vulnerable profile (e.g. use of sleep-inducing drugs, higher fear of surgery or anaesthesia) were at higher risk of worse emotional status towards the hospitalization during COVID-19 pandemic. Being operated in hospitals with lower surgical volume and with COVID-19 wards was associated with worse emotional status and fear of contagion.

**Conclusions:**

Additional fear and worse emotional status may be frequent in patients scheduled for elective surgery during COVID-19 pandemic. More than half of the participants to the survey were worried about not being able to receive family visits. Psychological support may be considered for patients at higher risk of psychological distress to improve perioperative wellbeing during the pandemic.

**Supplementary Information:**

The online version contains supplementary material available at 10.1186/s44158-021-00022-7.

## Background

Italy has been dramatically hit by COVID-19 [1]. During the earliest phases of the pandemic, most elective surgical activities and outpatients’ and chronic diseases services were suspended for months, until the national healthcare system succeeded to be restored in all of its components, as the restrictive measures allowed better management of the pandemic [2]. During later phases, these services have been profoundly modulated. However, the iterated changes regarding restrictive measures, the continuous updates on the number of contagions, along with contrasting opinions of the experts advertised on social media, may have contributed generating confusion, altered emotional status and fear of SARS-CoV-2 nosocomial contagions among common people. This was partly reflected by data showing diminished rates of admission and delayed presentation to the emergency wards for acute diseases, such as myocardial infarction, with outcomes worsening [3–5]. Furthermore, data exist on worsening outcomes in non-COVID-19 patients during the pandemic [6, 7]. To date, fragmented data exist on the extent of emotional and psychological distress generated by hospital admission during the pandemic in specific populations of patients, and no data exist on patients scheduled for surgery [8, 9].

The aim of this study was to evaluate the impact of the pandemic on emotional status and the fear of SARS-CoV-2 contagion in a cohort of elective surgical patients in Italy, scheduled for surgery during the COVID-19 pandemic.

## Methods

This study received approval from the Ethical Committee Palermo 2 on 14th December 2020 (318 AOR2020). The reporting of this study followed the Checklist for Reporting Of Survey Studies (CROSS) [10], which is available as Table S1 in the Additional file 1. The study was designed by the authors with insights from the Clinical Research Committee of the Italian Society of Anaesthesia, Analgesia and Intensive Care (*SIAARTI*) and received endorsement from the Society. The study period was from 12 January 2021 to 30 June 2021.

### Design and population

This was a multicentre nationwide prospective cross-sectional survey. All the anaesthesiologists registered to SIAARTI were invited to participate to the study, via emails and using the official newsletter and social media of the Society. Each centre could participate collecting data on up to a maximum of 100 patients, during a period no longer than 30 days.

All the adult patients scheduled to receive an elective surgical procedure in an operating room, under general or locoregional anaesthesia or sedation, were eligible. Both inpatients and outpatients were screened and eventually included during the anaesthesiologic pre-operative visit. Exclusion criteria were age inferior to 18 years old; urgent/emergency surgical procedures; being not mentally competent or already affected by a psychiatric disease with active symptoms (e.g. anxiety-depressive disorder). In case of eligibility, the questionnaire was administered during the same pre-operative visit. The time span between the visit and the surgery was not established a priori, and each centre followed its own internal protocols on anaesthesiologic pre-operative visits.

### Data collection

Data were collected using a questionnaire in the Italian language, administered either in a paper form or through a verbal interview in person, according to the patients’ preference. The questionnaire was composed of 28 closed questions, among which 24 regarded demographics, clinical history and surgical procedure and proposed anaesthesia. Six were specifically related to the COVID-19 pandemic in terms of temporal correlation with the surgical diagnosis, the effect on the emotional status towards the surgery, the fear of contagion during hospitalization and of being hospitalised without seeing family members due to restrictions. Four of these questions were considered as study outcome questions (see Table 2). All the questions were multiple-choice or forced 4 points Likert scale. The draft of the questionnaire was discussed among the authors until reaching consensus, and the drafted questions related to the emotional status and fear were then discussed and modified by a psychologist (FG), to improve content validity. All the authors approved the final version of the questionnaire. It was then implemented using REDCap (Research Electronic Data Capture) [11] by one of the authors (AN). A pilot test of both the questionnaire and the platform was performed by two authors independently (FM and MI). The original questionnaire is available as Additional file 2.

The data were collected anonymously by one or more investigators per centre. No specific training was provided to the local investigators but general rules for administration and data collection were provided. Before starting, the principal investigator of each centre completed a pre-study questionnaire reporting data on hospital characteristics, including surgical specialities, volume of surgery and eventual care for COVID-19 patients. The patients were asked to fill in the paper version of the questionnaire or to verbally answer the questions provided by the investigator. Study data were then recorded by the investigators using the REDCap hosted at *SIAARTI* data centre.

### Statistical analysis and sample size justification

After completing the data cleaning process, the data were analysed with descriptive statistics. Descriptive statistics included proportions for categorical and mean (standard deviation) for continuous variables. The amount of missing data was low (< 0.5%) and no assumptions were made for missing data.

We applied ordinal logistic regression models to evaluate variables independently associated with worse patients’ responses to the four study outcome questions: fear for routine check-ups, fear for SARS-CoV-2 infection during hospitalization, fear of hospitalization without seeing family members, worsening of the emotional status towards surgery due to COVID-19 pandemic. Results were reported as odds ratio (OR) with 95% confidence interval (CI). A stepwise approach was used to detect independent variables statistically significant in the multivariable models. This approach combines forward and backward selection methods in an iterative procedure (significance level of 0.05 both for entry and retention). Potential independent variables were patient characteristics (age, sex, education, marital status, number of children), presence of chronic diseases (cardiovascular, pulmonary, metabolic, oncological, immunological, other), cohabiting with chronic disease patients, use of sleep-inducing drugs, alcoholic beverages, drugs, isolation due to contact with COVID-19 patient, previous SARS-CoV-2 infection, family member with SARS-CoV-2 infection, surgery in the past and if it affected the current emotional status, type of anaesthesia (general-regional-sedation), type of patient (outpatient-inpatient), timepoint of surgery planning (before-during pandemic), fear of anaesthesia and surgical procedure, hospital characteristics (geographic area, number of beds, volume of surgeries per month, presence of COVID-19 ward, type of surgery procedure performed). For each ordinal logistic regression model, assumption of parallel lines was tested with Wald test for parallel lines and multicollinearity among variables was assessed by variance inflation factor (VIF). All *p* values were two-sided, with *p* values < 0.05 considered as statistically significant. Statistical analyses were performed with R, version 3.5.2 (The R Foundation for Statistical Computing, Vienna, Austria) and SAS software, version 9.4 (SAS Institute, Cary, NC, USA).

The sample size was estimated using a rule of thumb based on the number of independent variables in the models [12]. We estimated a sample size of 2350 patients to be included, for a total of 45 independent variables.

## Results

### Characteristics of centres, patients and surgical procedures

**Fig. 1:**
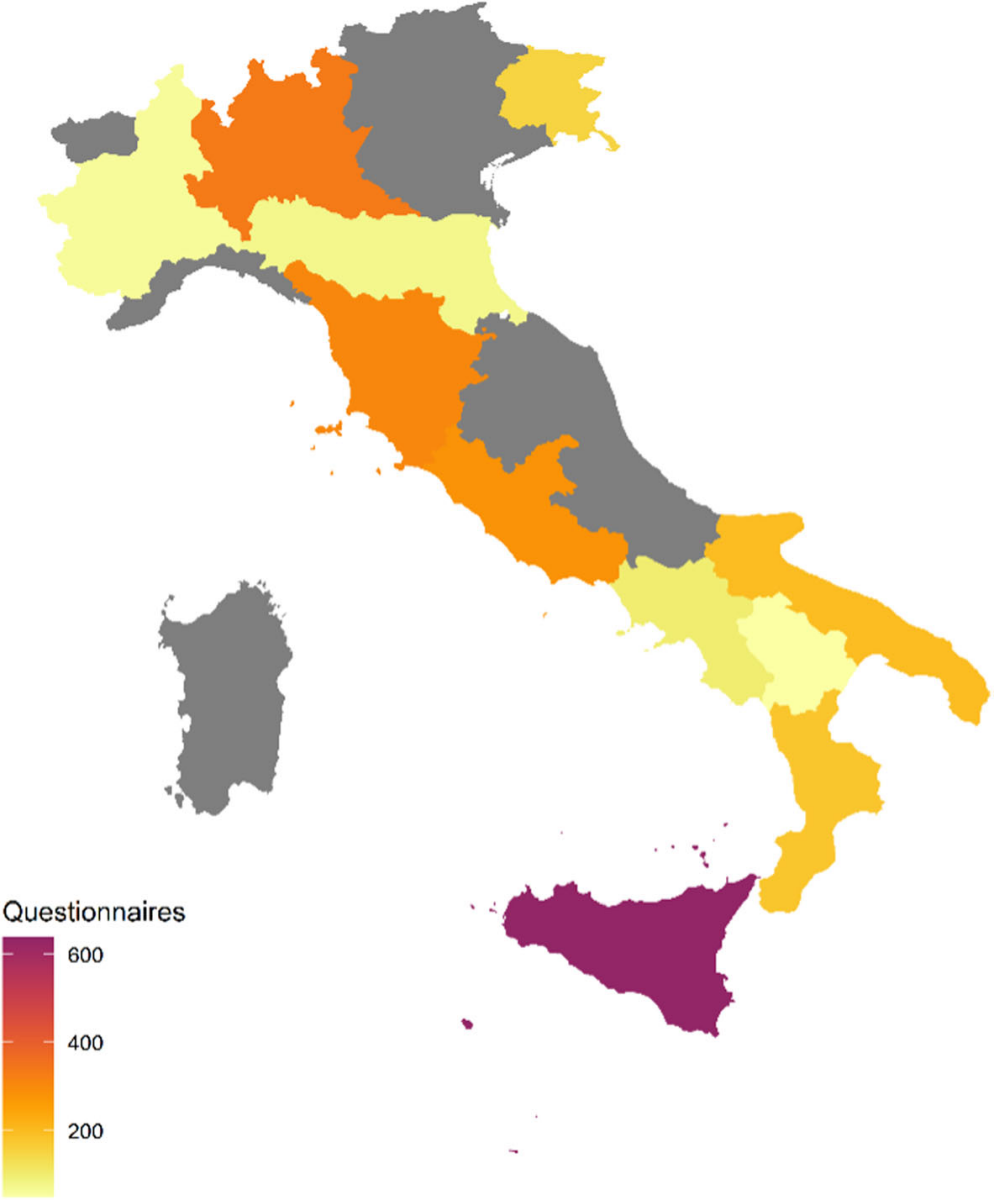
Geographical distribution of the centres in Italy and a colour legend reporting data on the number of provided questionnaires

A total of 29 Italian centres were involved in the study. The geographical distribution of the centres is available in Fig. 1. The characteristics of the centres are presented in Table S2 in the Additional file 1. Most of the centres reported volume of surgery counting more than 200 procedures per month (59%). Interestingly, 72% of the participating centres had at least one ward entirely dedicated to the care of patients with COVID-19. On a total of 7252 patients undergone to surgery during the study period (considering a fixed time of 4 weeks per centre and including urgent/emergency surgeries), 2376 patients were considered eligible and answered the questionnaire. The characteristics of the included patients are presented in Table 1. The population was gender-balanced, with 49.6% males and 50.4% females. The mean age was 58 years ± 16.61. Most of the patients were conjugated (80%) and had at least one child (78%). More than half of the surveyed patients had at least one chronic disease (54%), among which cardiovascular diseases were the commonest (58%). A low rate of patients declared the chronic use of alcohol (6%) or drug abuse (1%), but a higher percentage of patients declared the use of sleep-inducing drugs (15%). Only 6.7% of the surveyed patients had previously contracted a SARS-COV-2 infection, 17% had at least a relative who had a SARS-COV-2 infection and the 11% had got contact with someone positive to SARS-COV-2 and was put on precautionary isolation. The type of planned surgery was various, with the highest percentage of patients being evaluated prior to abdominal surgery (20%). The type of proposed anaesthesia was general anaesthesia in 64% of the cases.

**Table 1:** Characteristics of the surveyed patients

Questionnaires, *n*	2376
Age (years), mean ± sd	58.02 ± 16.61
Sex, *n* (%)	
Male	1177 (49.56)
Female	1198 (50.44)
Education, *n* (%)	
Primary school	858 (36.13)
Junior high school	480 (20.21)
Senior high school	337 (14.19)
Academic	700 (29.47)
Job description, *n* (%)	
Manager	49 (2.06)
Employee	432 (18.19)
Health worker	79 (3.33)
Self-employed	284 (11.96)
Worker	180 (7.58)
Homemaker	170 (7.16)
Student	33 (1.39)
Other	46 (1.94)
Unemployed	295 (12.42)
Retired	807 (33.98)
Spouse/partner, *n* (%)	
Yes	1895 (79.86)
No	478 (20.14)
Number of children, *n* (%)	
No children	517 (21.79)
1	498 (20.99)
2	909 (38.31)
3	326 (13.74)
> 3	123 (5.18)
Age of youngest child, *n* (%°)	
< 2	42 (2.26)
2–10	218 (11.75)
10–18	211 (11.37)
18–30	430 (23.18)
> 30	954 (51.43)
Cohabiting with chronic disease, *n* (%)	
Yes	357 (15.03)
No	2018 (84.97)
Affected by a chronic disease, *n* (%)	
Yes	1290 (54.32)
Cardiovascular	754 (58.45)
Pulmonary	217 (16.82)
Metabolic	464 (35.97)
Oncological	210 (16.28)
Immunological	116 (8.99)
Other	141 (10.93)
No	1085 (45.68)
Use of sleep-inducing drugs, *n* (%)	
No	2011 (84.75)
Yes, for years	191 (8.05)
Yes, for month	124 (5.23)
Yes, for weeks	47 (1.98)
Recurrent use of alcoholic beverages (6 months), *n* (%)	
Yes	139 (5.86)
No	2235 (94.14)
Recurrent drug abuse (6 months), *n* (%)	
Yes	27 (1.14)
No	2347 (98.86)
Isolation due to contact with COVID-19 patient, *n* (%)	
Yes	269 (11.33)
No	2106 (88.67)
Previous SARS-CoV-2 infection, *n* (%)	
Yes	158 (6.65)
No	2217 (93.35)
Family member with SARS-CoV-2 infection, *n* (%)	
Yes	409 (17.23)
No	1965 (82.77)
Other surgery in the past, *n* (%)	
Yes	1845 (77.72)
No	529 (22.28)
Previous surgery affects the current emotional status, *n* (%^)	
No	1024 (55.65)
Slightly	460 (25.00)
Moderately	284 (15.43)
Extremely	72 (3.91)
Type of surgery, *n* (%)	
Abdominal	478 (20.15)
Breast	162 (6.83)
Caesarean section	36 (1.52)
Cardiac/thoracic	240 (10.12)
Gynaecological	217 (9.15)
Neurological	58 (2.45)
Orthopaedic	175 (7.38)
Otolaryngology	181 (7.63)
Plastic	74 (3.12)
Urological	380 (16.02)
Vascular	170 (7.17)
Other	201 (8.47)
Type of anaesthesia, *n* (%)	
General	1519 (64.15)
Regional	615 (25.97)
Sedation	234 (9.88)
Type of patient, *n* (%)	
Outpatient	1265 (53.38)
Inpatient	1105 (46.62)

For all the questions, missing data were < 0.5%

°Percentage was calculated excluding 517 questionnaires reported “No child”

§Percentage was calculated excluding 1085 questionnaires reported “No chronic diseases”

^Percentage was calculated excluding 529 questionnaires reported “No surgery in the past”

### Outcomes

The relationship between patients’ emotional status and the pandemic SARS-COV-2 was specifically surveyed by four study outcome questions. An additional question was used to confirm that the patients attributed their emotional status to COVID-19 or to the procedure itself. The respondents were also asked to specify the first time they knew the need to undergo surgery, i.e. before or during the pandemic. The full results to these questions are presented in Table S2 in the Additional file 1.

The results to the four study outcome questions are shown in Fig. 2 and Table S3 in the Additional file 1. The results showed that 46% of the patients were at least slightly frightened of going to the hospital for routine checkups, 55% were afraid of getting SARS-CoV-2 infection during hospitalization and 62% declared fear of being hospitalised without seeing family members during the hospital stay. However, 50% of the patients declared that their emotional status with regards to the surgical procedure worsened due to COVID-19 pandemic and around 32% of the patients declared that the possibility of SARS-CoV-2 infection contributed, alone or in association to surgery/anaesthesia, as a main cause of the actual emotional status. Of note, 78% of the patients had precedent experiences of surgical procedures, but 56% declared that their emotional status was not influenced by these previous experiences.

**Figure:**
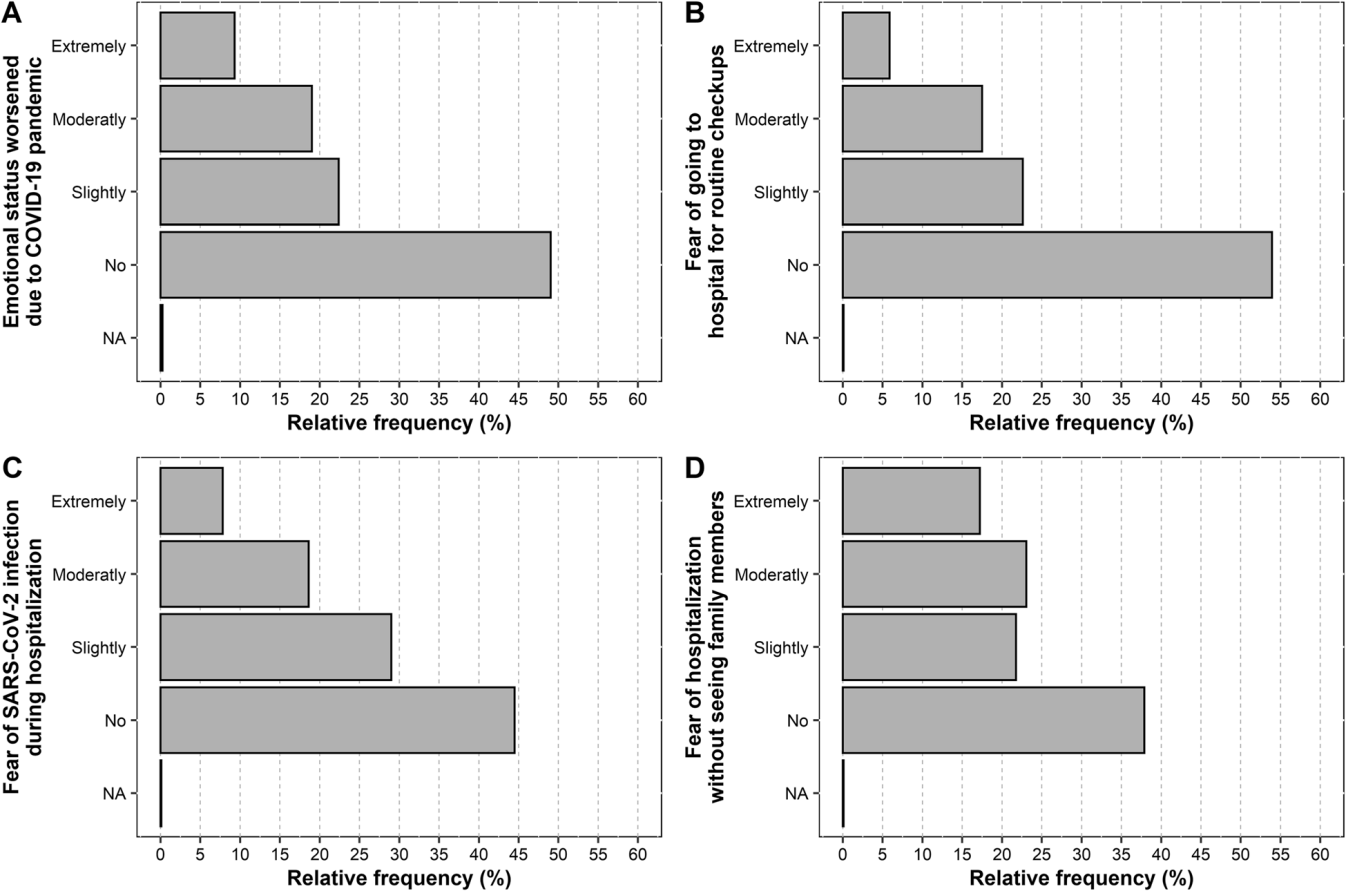
Fig. 2

### Adjusted analysis

The full results of the adjusted analysis are available in Table 2. The multivariable ordered logit models showed independent associations between several respondents’ characteristics, the type of anaesthesia and hospital-related factors and our study outcome questions.

**Table 2:** Multivariable ordered logit models

	Odds ratio (95% CI)	*p* value
Model 1—Dependent variable “Emotional status towards the surgical procedure worsened due to COVID-19 pandemic” *(N = 2361; Wald test of parallel lines assumption, p-value = 8454)*		
Spouse/partner (ref. no)	1.279 (1.041–1.572)	0.0191
Use of sleep-inducing drugs (ref. no.)	2.070 (1.657–2.587)	< .0001
Recurrent use of alcoholic beverages (6 months) (ref. no.)	0.636 (0.446–0.905)	0.0120
Previous SARS-CoV-2 infection (ref. no.)	0.607 (0.414–0.891)	0.0108
Isolation due to contact with COVID-19 patient (ref. no.)	1.577 (1.179–2.111)	0.0022
Fear of anaesthesia (ref. no)		
Slightly	1.447 (1.173–1.785)	0.0006
Moderately	2.070 (1.606–2.668)	< .0001
Extremely	2.363 (1.608–3.471)	< .0001
Fear of surgery (ref. no.)		
Slightly	1.655 (1.315–2.083)	< .0001
Moderately	3.065 (2.394–3.925)	< .0001
Extremely	3.151 (2.205–4.503)	< .0001
Previous surgery affects the current emotional status (ref. no. previous surgery)		
No	0.655 (0.526–0.815)	0.0002
Slightly	1.555 (1.222–1.979)	0.0003
Moderately	2.407 (1.825–3.177)	< .0001
Extremely	5.334 (3.189–8.922)	< .0001
Italian geographic area (ref. North)		
Centre	0.876 (0.680–1.128)	0.3042
South	1.525 (1.230–1.890)	0.0001
Hospital characteristics—performing multidisciplinary surgery (ref. no.)	1.843 (1.183–2.871)	0.0069
Volume of surgeries per month (ref. “< 100”)		
101–200	0.388 (0.276–0.546)	< .0001
> 200	0.350 (0.250–0.491)	< .0001
Hospital characteristics—presence of COVID-19 ward (ref. no.)	1.262 (1.028–1.551)	0.0265
Model 2—Dependent variable “Fear of going to hospital for routine check-ups” *(N = 2360; Wald test of parallel lines assumption, p value = 0.4373)*		
Male sex (ref. female)	0.691 (0.578–0.826)	0.0001
Spouse/partner (ref. no.)	1.271 (1.025–1.576)	0.0288
Use of sleep-inducing drugs (ref. no.)	1.828 (1.453–2.301)	< .0001
Previous SARS-CoV-2 infection (ref. no.)	0.498 (0.338–0.735)	0.0004
Family member with SARS-CoV-2 infection (ref. no.)	1.408 (1.107–1.790)	0.0053
Presence of chronic disease (ref. no.)	1.286 (1.071–1.543)	0.0069
Presence of immunological disease (ref. no.)	1.548 (1.055–2.271)	0.0254
Cohabiting with chronic disease patient (ref. no.)	1.326 (1.051–1.672)	0.0171
Type of patient (ref. outpatient)	1.210 (1.004–1.458)	0.0454
Previous surgery affects the current emotional status (ref. no. previous surgery)		
No	0.763 (0.608–0.957)	0.0194
Slightly	1.101 (0.856–1.416)	0.4547
Moderately	1.651 (1.246–2.188)	0.0005
Extremely	2.350 (1.402–3.939)	0.0012
Type of anaesthesia (ref. general)		
Locoregional	1.538 (1.260–1.876)	< .0001
Sedation	1.736 (1.313–2.294)	0.0001
Fear of anaesthesia (ref. no.)		
Slightly	1.356 (1.092–1.683)	0.0059
Moderately	2.181 (1.683–2.828)	< .0001
Extremely	2.793 (1.884–4.140)	< .0001
Fear of surgery (ref. no.)		
Slightly	1.835 (1.448–2.327)	< .0001
Moderately	2.790 (2.164–3.596)	<.0001
Extremely	2.052 (1.421–2.963)	0.0001
Italian geographic area (ref. north)		
Centre	0.863 (0.665–1.119)	0.2657
South	1.552 (1.226–1.964)	0.0003
Volume of surgeries per month (ref. “< 100”)		
101–200	0.332 (0.233–0.475)	< .0001
> 200	0.336 (0.239–0.474)	< .0001
Hospital characteristics—presence of COVID-19 ward (ref. no.)	1.315 (1.072–1.613)	0.0085
Model 3—Dependent variable “Fear of SARS-CoV-2 infection during hospitalization” *(N = 2362; Wald test of parallel lines assumption, p value = 0.4820)*		
Spouse/partner (ref. no.)		
Use of sleep-inducing drugs (ref. no.)	1.585 (1.271–1.976)	< .0001
Recurrent use of drugs (6 months) (ref. no.)	0.326 (0.137–0.774)	0.0111
Isolation due to contact with COVID-19 patient (ref. no.)	1.519 (1.140–2.023)	0.0043
Previous SARS-CoV-2 infection (ref. no.)	0.348 (0.236–0.513)	< .0001
Presence of pulmonary disease (ref. no.)	1.328 (1.017–1.735)	0.0373
Presence of oncological disease (ref. no.)	1.433 (1.093–1.879)	0.0093
Previous surgery affects the current emotional status (ref. no. previous surgery)		
No	0.948 (0.766–1.173)	0.6248
Slightly	1.371 (1.078–1.744)	0.0100
Moderately	1.922 (1.459–2.531)	< .0001
Extremely	3.304 (1.999–5.461)	< .0001
Type of anaesthesia (ref. general)		
Locoregional	1.229 (1.016–1.486)	0.0334
Sedation	1.777 (1.362–2.320)	< .0001
Fear of anaesthesia (ref. no.)		
Slightly	1.587 (1.297–1.943)	< .0001
Moderately	2.187 (1.707–2.801)	< .0001
Extremely	3.375 (2.303–4.947)	< .0001
Fear of surgery (ref. no.)		
Slightly	1.819 (1.459–2.269)	< .0001
Moderately	3.437 (2.707–4.364)	< .0001
Extremely	2.668 (1.879–3.789)	< .0001
Italian geographic area (ref. north)		
Centre	0.825 (0.648–1.049)	0.1156
South	1.487 (1.205–1.835)	0.0002
Volume of surgeries per month (ref. “< 100”)		
101–200	0.411 (0.292–0.580)	< .0001
> 200	0.472 (0.338–0.659)	< .0001
Hospital characteristics - presence of COVID-19 ward (ref. No)	1.328 (1.096–1.610)	0.0039
Model 4—Dependent variable “Fear of hospitalization without seeing family members” *(N = 2364; Wald test of parallel lines assumption, p value = 0.2282)*		
Sex (ref. female)	0.656 (0.557–0.773)	< .0001
Spouse/partner (ref. no)	1.487 (1.222–1.811)	0.0001
Use of sleep-inducing drugs (ref. no.)	1.586 (1.276–1.972)	< .0001
Previous SARS-CoV-2 infection (ref. no.)	0.615 (0.431–0.877)	0.0072
Family member with SARS-CoV-2 infection (ref. no.)	1.326 (1.054–1.668)	0.0158
Presence of oncological disease (ref. no.)	1.526 (1.168–1.995)	0.0020
Previous surgery affects the current emotional status (ref. no. previous surgery)		
No	0.820 (0.666–1.008)	0.0600
Slightly	1.308 (1.035–1.655)	0.0248
Moderately	1.713 (1.302–2.255)	0.0001
Extremely	2.206 (1.334–3.647)	0.0021
Fear of anaesthesia (ref. no.)		
Slightly	1.147 (0.941–1.399)	0.1748
Moderately	1.513 (1.183–1.935)	0.0010
Extremely	2.364 (1.627–3.434)	< .0001
Fear of surgery (ref. no.)		
Slightly	2.093 (1.695–2.586)	< .0001
Moderately	4.315 (3.406–5.467)	< .0001
Extremely	6.061 (4.237–8.669)	< .0001
Italian geographic area (ref. north)		
Centre	1.230 (0.974–1.553)	0.0824
South	1.432 (1.171–1.752)	0.0005
Hospital characteristics—performing multidisciplinary surgery (ref. no.)	1.677 (1.125–2.498)	0.0111
Volume of surgeries per month (ref. “< 100”)		
101–200	0.355 (0.252–0.501)	0.0000
> 200	0.380 (0.271–0.533)	< .0001

Among these factors, for example, having an oncological disease was independently associated with an increased risk of fear of SARS-CoV-2 infection during hospitalization and of being hospitalised without seeing family members. The use of sleep-inducing drugs and a higher level of fear towards both surgery and anaesthesia were associated with a worse emotional status and fear in all our outcome questions. Of note, a previous infection due to SARS-COV-2 was associated with a reduced risk of emotional distress or fear of SARS-CoV-2 infection during hospitalization. No association with the type of surgical procedures and our study outcomes was found; on the other hand, locoregional anaesthesia and sedation were associated with a higher level of fear of contagion during check-up visits and hospitalization. The presence of COVID wards in the hospital and a volume of surgery < 100 per month were associated with a worse emotional status due to COVID-19 pandemic and a higher risk of fear of SARS-CoV-2 contagion.

## Discussion

To the best of our knowledge, this is the first study specifically addressing the emotional status of elective surgical patients during the pandemic COVID-19. The main finding of our study is that one out of two patients scheduled for elective surgery may be frightened of attending routine checkups and of getting infected during hospitalization. Furthermore, even more than half of the patients were frightened of spending the entire period of hospitalization being prevented from receiving visits by their relatives. Globally, these data suggest an important additional trigger for stress and worse emotional status due to the current pandemic situation in patients scheduled for elective surgery, independently from patients’ characteristics and surgical factors.

These findings were in line with similar studies, recently conducted in different populations of patients. Indeed, a recent survey has recently shown that 65% of a cohort of 156 patients with lung cancers felt relieved, in terms of feeling a reduced risk of SARS-COV-2 contagion, when the oncologist cancelled their treatment/visit due to the pandemic [13]. Moreover, the decrease of admissions to emergency departments and hospitalizations during the early phases of the pandemic has been measured and described [14]. The authors showed that the reduction encompassed all the pathological conditions, including time-dependent ones, and that it started earlier than the local transmission, suggesting that such population response was likely more affected by the national level authority risk message than the real situation [14]. It can be argued that many modifiable factors may have contributed to this scenario, such as the confusing and sometimes contrasting communication promoted by social media on the topic of pandemic and contagions [15], or the efficacy of safety measures adopted by the hospitals in the most overwhelming periods of the pandemic [16].

Specific categories of patients may be at a higher risk of altered emotional status during the pandemic, as shown by our adjusted analysis. We identified patient-related factors variably associated with worse emotional status or fear, such as being affected by chronic, oncological or immunological diseases, cohabiting with a relative with chronic disease, or being conjugated. Interestingly, patients who showed most emotionally vulnerable profiles (e.g. those who chronically took sleep-inducing drugs, those who declared to feel feared also due to the surgery and the anaesthesia and those whose previous surgical experience worsened the current emotional status) were at higher risk for a negative emotional status towards the hospitalization during COVID-19 pandemic.

Different associations were found between our study outcomes and the national geographic locations. This may be explained by the different situations of the pandemic among the north, centre and south of Italy during the study period with different psychological impact of people who needed surgery.

Our data contribute to discuss that it is probably worth to specifically address the modifiable factors and identify the patients at the highest risk of emotional distress during the pandemic period, so that countermeasures can be taken appropriately. The provision of professional psychological support to the most vulnerable categories of patients could be of help, together with tailored communication campaigns, aiming to reduce the effects of fear in the worst period of the pandemic. The association between the presence of COVID wards in the hospital and worse study outcomes also deserves attention. This issue can be addressed informing patients about infection control strategies, differentiated pathways and other safety measures adopted by hospitals treating both COVID-19 and non-COVID-19 patients.

Our study has limitations. First, the questionnaire was not subjected to any formal validation and no validated tool was used to measure the extent of the emotional distress and fear of patients. This was mainly due to the nature of our research question, directly related to the period of the pandemic and, thus, situational. Second, the design of the study is explorative *per se*, and caution is needed when considering the results. We did not follow patients later on during the hospitalization, and no associations with subsequent clinical outcomes or psychological status were assessed. We did not consider time to surgery in our analysis, as we anticipated that the date of the surgery could not be certainly set in a relevant proportion of patients at the time of pre-operative anaesthesiologic visit during the pandemic. We also did not collect any anthropometric data (e.g. BMI). The external validity of our results is limited outside the Italian country. We could not provide a response rate as per the definition. However, the sample reached may be considered as representative if compared with the number of patients undergoing surgery during the period of study in the involved centres (four weeks per centre), from which urgent/emergency cases were excluded, together with those exceeding the limit of 100 patients per centre.

Moreover, we did not collect data on the vaccination status of the respondents or on the effect of vaccine availability on the study outcomes, as at the protocol stage, no vaccination campaign was available or publicly planned. The current availability of vaccination and the less crowded condition of hospitals could make our results already outdated. On the other hand, the pandemic is not over, and there are many uncertainties on the need for the third shot of vaccine and on SARS-COV-2 variants.

The study has strengths, such as the large number of respondents from many different centres in different regions of the country, and the very low extent of missing data. Moreover, our study cohort seems to be representative of the general elective surgery population of high-income countries, considering the size and the general characteristics. The use of an easy-to-comprehend 4-points Likert scale for the outcome questions, forcing the respondents to avoid a neutral evaluation, made the question answering process easier and focused.

## Conclusions

Basing on our data, additional fear and worse emotional status may be frequent in patients scheduled for elective surgery during COVID-19 pandemic. More than half of the participants to the survey were worried about not being able to receive family visits. Psychological support may be considered to help patients scheduled for surgery to overcome this worse emotional status and to improve perioperative wellbeing during the pandemic.

## Supplementary Information

**Additional file 1: Table S1.** Checklist for Reporting Of Survey Studies (CROSS): **Table S2.** characteristics of the centres; **Table S3.** Full responses to study outcome questions and other relevant questions

**Additional file 2:.** Original Italian questionnaire

## Data Availability

The datasets used and/or analysed during the current study are available from the corresponding author on reasonable request.

## Abbreviations

COVID-19: Coronavirus disease 2019
CROSS: Checklist for Reporting Of Survey Studies
ED: Emergency ward
SIAARTI: Italian Society of Anaesthesia, Analgesia and Intensive Care
OR: Odds ratio
CI: Confidence interval
