# Supplementary material for: Emotional status and fear in patients scheduled for elective surgery during COVID-19 pandemic: a nationwide cross-sectional survey (COVID-SURGERY)
Source: J Anesth Analg Crit Care. 2021 Nov 25;1:17. doi: 10.1186/s44158-021-00022-7 (PMC8613518; doi:10.1186/s44158-021-00022-7)
Supplement: Supplementary file 1 — Additional file 1: Table S1. Checklist for Reporting Of Survey Studies (CROSS): Table S2. characteristics of the centres; Table S3. Full responses to study outcome questions and other relevant questions [file 44158_2021_22_MOESM1_ESM.docx]

Additional File to:

**Emotional status and fear in patients scheduled for**

**elective surgery during COVID-19 pandemic:**

**A nationwide cross-sectional survey (COVID-SURGERY)**

Francesca Montalto^1§^, Mariachiara Ippolito^2§^, Alberto Noto^3^, Fabiana Madotto^4^, Filippa Gelardi^5^, Paolino Savatteri^1^, Antonino Giarratano^2,6^, Andrea Cortegiani^2,6^ and the *SIAARTI Study Group**

^§^Francesca Montalto and Mariachiara Ippolito are shared first authors

^1^ UOC Anestesia Rianimazione 1 PO Villa Sofia AOOR Villa Sofia-Cervello

^2^Department of Surgical Oncological and oral Science (Di.Chir.On.S.), University of Palermo, Italy

^3^ Department of Human Pathology of the Adult and Evolutive Age "Gaetano Barresi", Division of Anesthesia and Intensive Care, University of Messina, Messina, Italy.

^4^ Value-Based Healthcare Unit, IRCCS Multimedica, 20099 Sesto San Giovanni, Milan, Italy

^5^Freelance psychologist

^6^Department of Anesthesia, Intensive Care and Emergency, Policlinico Paolo Giaccone, Palermo, Italy

*Corresponding author: Prof. Andrea Cortegiani. Department of Surgical, Oncological and Oral Science (Di.Chir.On.S.), University of Palermo, Italy. Department of Anaesthesia, Intensive Care and Emergency, Policlinico Paolo Giaccone, Palermo, Italy, Via del Vespro 129, 90127 Palermo, Italy. Email: andrea.cortegiani@unipa.it; Phone: +390916552751

**List of investigators of the *SIAARTI Study Group***

- Centro di Riferimento Oncologico di Aviano – IRCCS (Aviano – Pordenone): Fabrizio Brescia, Fabio Fabiani, Chiara Zanier, Elisa Nadalini;
- Spedali Civili Brescia (Brescia): Eros Gambaretti;
- IRCCS “de Bellis” (Castellana Grotte): Francesco Gabriele;
- Azienda Ospedaliero Universitaria Policlinico "G.Rodolico - San Marco" (Catania): Marinella Astuto, Paolo Murabito, Filippo Sanfilippo;
- Fondazione Istituto "G. Giglio" (Cefalù): Giovanni Misseri; Alessandra Moscarelli
- Azienda ospedaliera universitaria di Ferrara - Università di Ferrara (Ferrara): Savino Spadaro, Enrico Bussolati, Eleonora Squadrani;
- Azienda Ospedaliero-Universitaria Careggi (Firenze): Gianluca Villa, Raffaella d'Errico, Giulia Cocci, Iacopo Lanini;
- Azienda mista ospedaliero Universitaria OORR- Università di Foggia (Foggia): Lucia Mirabella, Alessandra Morelli, Livio Tullo, Girolamo Caggianelli;
- Università degli Studi di Genova (Genova): Lorenzo Ball;
- Azienda Ospedaliera Universitaria Gaetano Martino (Messina): Margherita Iiriti; Francesca Giordani; Massimiliano Giardina; Anna Teresa Mazzeo.
- Fondazione IRCCS Ca' Granda Ospedale Maggiore Policlinico di Milano (Milano): Giacomo Grasselli, Emanuele Cattaneo, Salvatore Alongi, Cristina Marenghi;
- IRCCS San Raffele Scientific Institute (Milano): Marilena Marmiere, Margherita Rocchi, Stefano Turi, Giovanni Landoni;
- ASST Grande Ospedale Metropolitano Niguarda, Department of Medicine and Surgery, University of Milan-Bicocca (Monza): Vito Torrano, Giulia Tinti, Antonio Giorgi, Roberto Fumagalli;
- Ospedale Regina Montis Regalis Mondovì (Mondovì): Francesco Salvo, Ilaria Blangetti;
- Istituto Tumori Napoli (Napoli): Marco Cascella, Cira Antonietta Forte;
- Università degli Studi di Padova: Paolo Navalesi;
- Villa Sofia-Cervello, Anestesia Rianimazione 1 (Palermo): Marta Montalbano, Valentina Chiarelli, Giuseppe Bonanno, Francesco Paolo Ferrara;
- Villa Sofia-Cervello, Neurorianimazione (Palermo): Innocenza Pernice;
- Policlinico Paolo Giaccone, Anestesia e Rianimazione e Terapia del Dolore (Palermo): Giulia Catalisano, Claudia Marino, Gabriele Presti, Dario Calogero Fricano;
- A.O.U.P. Paolo Giaccone, Cardioanestesia e Terapia Intensiva Post-Operatoria Cardio-Toraco-Vascolare (Palermo): Rosa Fucà, Cesira Palmeri Di Villalba;
- ARNAS Ospedali Civico Di Cristina Benfratelli (Palermo): Maria Teresa Strano, Sabrina Caruso, Antonino Scafidi, Vincenzo Mazzarese;
- Maria Eleonora Hospital (Palermo): Ettore Augugliaro, Valeria Terranova;
- Azienda Ospedaliero Universitaria Pisana, Unità di Anestesia e Rianimazione Interdipartimentale (Pisa): Francesco Forfori, Francesco Corradi, Erika Taddei, Alessandro Isirdi;
- Azienda Ospedaliero Universitaria Pisana, UO Anestesia e Rianimazione Trapianti (Pisa): Giorgia Pratesi, Francesca Puccini;
- San Carlo Hospital Potenza Italy (Potenza): Gianluca Paternoster, Alessio Barile;
- G.O.M. Grande Ospedale Metropolitano Bianchi- Melacrino -Morelli, U.O.C. Terapia Intensiva e Anestesia (Reggio Calabria): Marco Tescione, Irene Santacaterina, Eliana Maria Siclari;
- G.O.M. Grande Ospedale Metropolitano "Bianchi-Melacrino-Morelli", UO Cardioanestesia e Rianimazione Cardiochirurgica (Reggio Calabria): Vincenzo Francesco Tripodi, Mariacristina Vadalà;
- Campus Biomedico di Roma (Roma): Felice Eugenio Agrò, Giuseppe Pascarella, Chiara Piliego
- Policlinico Universitario Fondazione Agostino Gemelli (Roma): Paola Aceto, Gennaro De Pascale, Alessandra Dottarelli, Bruno Romanò, Andrea Russo;
- Istituto Nazionale Tumori Regina Elena IRCCS (Roma): Marco Covotta, Valeria Giorgerini, Federica Sardellitti, Giulia Maria Vitelli;
- Università La Sapienza di Roma (Roma): Flaminia Coluzzi;
- Azienda Sanitaria Universitaria Friuli Centrale (Udine): Tiziana Bove, Luigi Vetrugno;

**Content.**

**Table S1. Checklist for Reporting Of Survey Studies (CROSS)**

**Table S2. Characteristics of participating centers.**

**Table S3. Responses to the six questions related to COVID-19 pandemic**

**Table S1. Checklist for Reporting Of Survey Studies (CROSS)**

| **Section/topic** | **Item** | **Item description** | **Reported on page #** |
| --- | --- | --- | --- |
| **Title and abstract** | | |  |
| Title and abstract | 1a | State the word “survey” along with a commonly used term in title or abstract to introduce the study’s design. | 1 |
|  | 1b | Provide an informative summary in the abstract, covering background, objectives, methods, findings/results, interpretation/discussion, and conclusions. | 2 |
| **Introduction** | | |  |
| Background | 2 | Provide a background about the rationale of study, what has been previously done, and why this survey is needed. | 4 |
| Purpose/aim | 3 | Identify specific purposes, aims, goals, or objectives of the study. | 4 |
| **Methods** | | |  |
| Study design | 4 | Specify the study design in the methods section with a commonly used term (e.g., cross-sectional or longitudinal). | 5 |
|  | 5a | Describe the questionnaire (e.g., number of sections, number of questions, number and names of instruments used). | 5 |
| Data collection methods | 5b | Describe all questionnaire instruments that were used in the survey to measure particular concepts. Report target population, reported validity and reliability information, scoring/classification procedure, and reference links (if any). | 5 |
|  | 5c | Provide information on pretesting of the questionnaire, if performed (in the article or in an online supplement). Report the method of pretesting, number of times questionnaire was pre-tested, number and demographics of participants used for pretesting, and the level of similarity of demographics between pre-testing participants and sample population. | 5 |
|  | 5d | Questionnaire if possible, should be fully provided (in the article, or as appendices or as an online supplement). | 5 |
| Sample characteristics | 6a | Describe the study population (i.e., background, locations, eligibility criteria for participant inclusion in survey, exclusion criteria). | 5 |
|  | 6b | Describe the sampling techniques used (e.g., single stage or multistage sampling, simple random sampling, stratified sampling, cluster sampling, convenience sampling). Specify the locations of sample participants whenever clustered sampling was applied. | 5 |
|  | 6c | Provide information on sample size, along with details of sample size calculation. | NA |
|  | 6d | Describe how representative the sample is of the study population (or target population if possible), particularly for population-based surveys. | 5 |
| Survey  administration | 7a | Provide information on modes of questionnaire administration, including the type and number of contacts, the location where the survey was conducted (e.g., outpatient room or by use of online tools, such as SurveyMonkey). | 5-6 |
|  | 7b | Provide information of survey’s time frame, such as periods of recruitment, exposure, and follow-up days. | 5 |
|  | 7c | Provide information on the entry process:  –>For non-web-based surveys, provide approaches to minimize human error in data entry.  –>For web-based surveys, provide approaches to prevent “multiple participation” of participants. | 5-6 |
| Study preparation | 8 | Describe any preparation process before conducting the survey (e.g., interviewers’ training process, advertising the survey). | 5-6 |
| Ethical considerations | 9a | Provide information on ethical approval for the survey if obtained, including informed consent, institutional review board [IRB] approval, Helsinki declaration, and good clinical practice [GCP] declaration (as appropriate). | 5 |
|  | 9b | Provide information about survey anonymity and confidentiality and describe what mechanisms were used to protect unauthorized access. | 6 |
| Statistical  analysis | 10a | Describe statistical methods and analytical approach. Report the statistical software that was used for data analysis. | 6 |
|  | 10b | Report any modification of variables used in the analysis, along with reference (if available). | 6 |
|  | 10c | Report details about how missing data was handled. Include rate of missing items, missing data mechanism (i.e., missing completely at random [MCAR], missing at random [MAR] or missing not at random [MNAR]) and methods used to deal with missing data (e.g., multiple imputation). | 6 |
|  | 10d | State how non-response error was addressed. | 6 |
|  | 10e | For longitudinal surveys, state how loss to follow-up was addressed. | NA |
|  | 10f | Indicate whether any methods such as weighting of items or propensity scores have been used to adjust for non-representativeness of the sample. | NA |
|  | 10g | Describe any sensitivity analysis conducted. | NA |
| **Results** | | |  |
| Respondent characteristics | 11a | Report numbers of individuals at each stage of the study. Consider using a flow diagram, if possible. | 6 |
|  | 11b | Provide reasons for non-participation at each stage, if possible. | NA |
|  | 11c | Report response rate, present the definition of response rate or the formula used to calculate response rate. | 6 |
|  | 11d | Provide information to define how unique visitors are determined. Report number of unique visitors along with relevant proportions (e.g., view proportion, participation proportion, completion proportion). | NA |
| Descriptive  results | 12 | Provide characteristics of study participants, as well as information on potential confounders and assessed outcomes. | 6-8 |
| Main findings | 13a | Give unadjusted estimates and, if applicable, confounder-adjusted estimates along with 95% confidence intervals and p-values. | Figure 2, Table S3, Table 2 |
|  | 13b | For multivariable analysis, provide information on the model building process, model fit statistics, and model assumptions (as appropriate). | 6 |
|  | 13c | Provide details about any sensitivity analysis performed. If there are considerable amount of missing data, report sensitivity analyses comparing the results of complete cases with that of the imputed dataset (if possible). | NA |
| **Discussion** | | |  |
| Limitations | 14 | Discuss the limitations of the study, considering sources of potential biases and imprecisions, such as non-representativeness of sample, study design, important uncontrolled confounders. | 10 |
| Interpretations | 15 | Give a cautious overall interpretation of results, based on potential biases and imprecisions and suggest areas for future research. | 8-10 |
| Generalizability | 16 | Discuss the external validity of the results. | 10 |
| **Other sections** | | |  |
| Role of funding source | 17 | State whether any funding organization has had any roles in the survey’s design, implementation, and analysis. | 18 |
| Conflict of interest | 18 | Declare any potential conflict of interest. | 18 |
| Acknowledgements | 19 | Provide names of organizations/persons that are acknowledged along with their contribution to the research. | 18-19 |

**Table S2. Characteristics of participating centers.**

|  | N (%) |
| --- | --- |
| N | 29 |
| Institution |  |
| Private | 5 (17.24) |
| Public | 24 (82.76) |
| Beds per institution |  |
| < 250 | 5 (17.24) |
| 250-500 | 4 (13.79) |
| 501-750 | 5 (17.24) |
| > 750 | 15 (51.72) |
| Type of surgery procedures |  |
| Single | 2 (6.90) |
| Multi | 27 (93.10) |
| Institution with COVID-19 wards |  |
| No | 8 (27.59) |
| Yes | 21 (72.41) |
| Volume of surgeries per month |  |
| < 50 | 1 (3.45) |
| 51-100 | 2 (6.90) |
| 101-200 | 9 (31.03) |
| >200 | 17 (58.62) |

**Table S3. Responses to the six questions related to COVID-19 pandemic**

| Fear of going to the hospital for routine checkups, n (%) |  |
| --- | --- |
| No | 1,281 (53.96) |
| Slightly | 537 (22.62) |
| Moderately | 416 (17.52) |
| Extremely | 140 (5.90) |
| Fear of SARS-CoV-2 infection during hospitalization, n (%) |  |
| No | 1,057 (44.52) |
| Slightly | 689 (29.02) |
| Moderately | 442 (18.62) |
| Extremely | 186 (7.83) |
| Fear of hospitalization without seeing family members, n (%) |  |
| No | 900 (37.91) |
| Slightly | 517 (21.78) |
| Moderately | 548 (23.08) |
| Extremely | 409 (17.23) |
| Emotional status towards surgery due to COVID-19 pandemic, n (%) |  |
| No | 1,165 (49.16) |
| Slightly | 532 (22.45) |
| Moderately | 452 (19.07) |
| Extremely | 221 (9.32) |
| When I found out about the surgery, n (%) |  |
| Before COVID-19 Pandemic | 230 (9.70) |
| During COVID-19 Pandemic | 2,141 (90.30) |
| Main cause of the emotional state, n (%) |  |
| SARS-CoV-2 Infection | 228 (9.61) |
| Surgery / Anesthesia | 789 (33.26) |
| Both | 538 (22.68) |
| None | 817 (34.44) |

Data are reported as numbers and percentages
