# Supplementary material for: Emotional status and fear in patients scheduled for elective surgery during COVID-19 pandemic: a nationwide cross-sectional survey (COVID-SURGERY)
Source: J Anesth Analg Crit Care. 2021 Nov 25;1:17. doi: 10.1186/s44158-021-00022-7 (PMC8613518; doi:10.1186/s44158-021-00022-7)
Supplement: Supplementary file 2 — Additional file 2:. Original Italian questionnaire [file 44158_2021_22_MOESM2_ESM.pdf]

Scheda Paziente

Record ID	__________________
Utente del Centro	__________________ (Utente che registra la scheda)
Data dell'intervento chirurgico	__________________ (programmata o eseguita)
Età	__________________ (Età espressa in anni)
Sesso	◯ M ◯ F
Titolo di studio	◯ Licenza elementare<br>◯ Licenza media<br>◯ Diploma<br>◯ Laurea
Attuale impiego	◯ Operaio<br>◯ Impiegato<br>◯ Libero professionista<br>◯ Dirigente<br>◯ Operatore sanitario<br>◯ Disoccupato<br>◯ Pensionato<br>◯ Casalinga<br>◯ Altro
Specificare altro impiego	__________________
Sposato o convivente?	◯ Yes ◯ No
Numero di figli	__________________
Età del figlio piu' piccolo	◯ < 2 anni<br>◯ 2 - 10<br>◯ 10 - 18<br>◯ 18 - 30<br>◯ > 30
Convive con persone affette da patologie croniche, che necessitano di assistenza (anche parzialmente)?	◯ Yes ◯ No
Soffre di patologie croniche?	◯ Yes ◯ No

Specificare il tipo di patologia	☐ Cardiovascolari<br>☐ Polmonari<br>☐ Metaboliche (Diabete, endocrine etc)<br>☐ Oncologiche<br>☐ Autoimmuni<br>☐ Altro
Specificare altro	__________________________
Assume farmaci che la aiutano a dormire?	◯ Si, da qualche mese<br>◯ Si, da qualche settimana<br>◯ Si, da anni<br>◯ No
Durante gli ultimi sei mesi, ha fatto uso ricorrente di alcolici?	◯ Yes<br>◯ No
Durante gli ultimi sei mesi, ha fatto uso ricorrente di droghe?	◯ Yes<br>◯ No
E' mai stato in isolamento per contatto con soggetti positivi a COVID-19?	◯ Yes<br>◯ No
E' mai stato positivo per COVID-19?	◯ Yes<br>◯ No
Ha mai avuto familiari o conviventi positivi al COVID-19?	◯ Yes<br>◯ No
E' stato sottoposto in precedenza ad altri interventi chirurgici?	◯ Yes<br>◯ No

	1 - No	2 - Poco	3 - Abbastanza	4 - Molto
Ritengo che il mio attuale stato emotivo rispetto all'intervento chirurgico sia peggiorato a causa della mia precedente esperienza chirurgica?	◯	◯	◯	◯

Indichi il tipo di intervento chirurgico a cui dovrà sottoporsi:	◯ Neurochirurgico<br>◯ Cardiochirurgico / Toracico<br>◯ Ch. Vascolare<br>◯ Ch. Mammella<br>◯ Ch. Plastica<br>◯ Ch. Addominale<br>◯ Parto Cesareo<br>◯ Ortopedico<br>◯ Urologico<br>◯ Ginecologico<br>◯ Otorinolaringoiatrico<br>◯ Altro
Specificare altro	__________________________

Che tipo di anestesia le è stata proposta per il suo intervento?	◯ Generale<br>◯ Loco-Regionale (es. spinale, peridurale, blocco di uno o più nervi)<br>◯ Sedazione
Attualmente lei si trova	◯ Ricoverato presso un reparto di questo ospedale<br>◯ Accedo oggi in ospedale per visita ambulatoriale

In relazione alla pandemia da COVID-19, esprima le risposte alle seguenti domande:

	1 - No	2 - Poco	3 - Abbastanza	4 - Molto
Ho paura di recarmi in ospedale per eseguire controlli di routine?	◯	◯	◯	◯
Ho paura di potermi contagiare con il virus durante l'intervento e la degenza?	◯	◯	◯	◯
Ho paura di dover affrontare la degenza senza possibilità di ricevere visite dai miei familiari?	◯	◯	◯	◯
Ritengo che il mio stato emotivo rispetto all'intervento chirurgico sia peggiorato a causa della pandemia COVID-19?	◯	◯	◯	◯

Ho saputo di dovermi sottoporre ad intervento chirurgico:	◯ Prima della pandemia<br>◯ Durante la pandemia
Ritengo che il mio stato emotivo sia influenzato principalmente da:	◯ Paura del contagio da COVID-19<br>◯ Paura dell'intervento chirurgico / anestesia<br>◯ Entrambe in egual misura<br>◯ Non ritengo di avere paura di nessuna delle due
